# Selective loss of primary cilia and neurotrophic signaling in G51D α-synuclein mice highlights a common pathway to Parkinson’s disease

**DOI:** 10.1073/pnas.2619797123

**Published:** 2026-08-10

**Authors:** Yu-En Lin, Ebsy Jaimon, YoungDoo Kim, Annabeth Loftman, Aaran Vijayakumaran, Benjamin D. W. Belfort, Claire Y. Chiang, Benjamin R. Arenkiel, Huda Y. Zoghbi, Suzanne R. Pfeffer

**Affiliations:** ^a^ Department of Biochemistry, Stanford University School of Medicine, Stanford, CA 94305; ^b^ Aligning Science Across Parkinson’s Collaborative Research Network, Chevy Chase, MD 20815; ^c^ Department of Molecular and Human Genetics, Baylor College of Medicine, Houston, TX 76798; ^d^ Jan and Dan Duncan Neurological Research Institute at Texas Children’s Hospital, Houston, TX 76798; ^e^ Department of Neuroscience, Baylor College of Medicine, Houston, TX 76798; ^f^ Department of Neurology and Pediatrics, Baylor College of Medicine, Houston, TX 76798; ^g^HHMI, Chevy Chase, MD 20815

**Keywords:** Parkinson’s disease, neurotrophic signaling, alpha-Synuclein, primary cilia

## Abstract

Parkinson’s disease is characterized by dopaminergic neuron loss and accumulation of α-synuclein aggregates in the brain. G51D α-synuclein knock-in mice provide a genetically and clinically relevant model of disease, exhibiting early olfactory deficits, age-dependent motor impairment, and progressive phospho-α-synuclein accumulation. In multiple Parkinson’s disease models, striatal cholinergic and parvalbumin interneurons, as well as astrocytes, lose primary cilia and the neurotrophic signaling needed to sustain dopaminergic neurons. We show here that G51D α-synuclein mice share these phenotypes. Phospho-Ser129 α-synuclein accumulation correlates with cilia loss in cholinergic interneurons but not in spiny projection neurons that accumulate higher phospho-α-synuclein levels. In the piriform cortex, parvalbumin neurons lose primary cilia and downregulate Neurturin, potentially contributing to olfactory dysfunction. Within the peripheral olfactory epithelium, horizontal basal cells lose cilia, whereas multiciliated olfactory sensory neuron cilia remain intact. These findings reveal convergent cellular vulnerabilities across Parkinson’s disease models and highlight a pathogenic role for impaired ciliary signaling.

## Significance

Parkinson’s disease is caused in part by the toxic accumulation of α-synuclein, which damages brain cells that produce dopamine. Using mice carrying a Parkinson’s-associated α-synuclein mutation, we show that α-synuclein buildup is linked to the loss of primary cilia in specific cell types in the striatum and olfactory pathway. These findings align with results from other genetic and idiopathic Parkinson’s disease models, suggesting that disrupted ciliary signaling is a common feature of the disease and a potential target for future therapies.

Most Parkinson’s disease (PD) is of unknown cause, although pesticides and other environmental insults are strongly implicated (1). Hallmarks of PD include resting tremor, rigidity, and bradykinesia due to death of dopaminergic neurons in the substantia nigra that govern movement coordination (1). Loss of smell and constipation can precede these symptoms by more than 10 y, along with rapid eye movement (REM) sleep behavior disorder in which a person acts out their dreams because normal, REM-related muscle paralysis is absent. Abnormal aggregation of alpha-synuclein (α-Syn) is a key contributor to PD, promoting neurodegeneration through toxic oligomers and fibrils that disrupt synaptic function, membrane integrity, and cellular homeostasis (2, 3). Misfolded α-Syn spreads between neurons, leading to so-called Lewy pathology (4, 5).

10 to 15% of PD cases are caused by identifiable genetic mutations or genetic risk variants, including missense mutations in *SNCA* encoding α-Syn, activating mutations in the Leucine Rich Repeat Kinase 2 (LRRK2) or loss of function mutations in *GBA1* encoding lysosomal glucocerebrosidase (6). In our efforts to understand the molecular basis of LRRK2-PD, we showed that LRRK2 phosphorylation of Rab10 protein blocks primary cilia formation in cell culture by a process that requires the phosphoRab effector protein, RILPL1 (7, 8). In the striatum of mice harboring pathogenic LRRK2 mutations (G2019S, R1441C) or lacking the genes encoding either PPM1H or PPM1M phosphatases that reverse LRRK2 action (9, 10), we have shown that primary cilia are lost in a cell-type specific manner: Rare, cholinergic (ChAT) and parvalbumin (PV) neurons and ALDH1L1^+^ or GFAP^+^ astrocytes lose their cilia, while the much more abundant spiny projection neurons do not (8, 10–14). Cell type specificity of cilia loss is not dictated by overall LRRK2 or PPM1H expression levels (12). However, among ChAT neurons, higher LRRK2 levels correlate with decreased ciliation (12).

Primary cilia are essential for Hedgehog signaling (15), which is critical for the survival of nigral dopamine neurons that project to the dorsal striatum and the cholinergic neurons located there (16). We have shown that cilia loss correlates with loss of Hedgehog signaling in the striatum (8), and the concomitant loss of cilia-dependent transcription of neurotrophic factors that support dopamine neurons (12–14). Thus, in the mouse striatum, cilia loss decreases production of glial-derived neurotrophic factor (GDNF) by ChAT neurons (12), Neurturin (NRTN) by PV neurons (13), and Brain-derived neurotrophic factor (BDNF) by astrocytes (17). Loss of these factors leads to decreased density of the dopaminergic axonal arbor in the striatum, as monitored by loss of tyrosine hydroxylase or GDNF Receptor Alpha-1 staining (14). The importance of Hedgehog signaling is recapitulated in *Gba1* mutant mice that retain their cilia but show decreased Hedgehog signaling because of an altered lipid composition in their otherwise normal-appearing cilia (17).

Unlike most idiopathic PD, ~30% of LRRK2-PD lack classical Lewy body pathology (18). Because of this, it was not clear whether LRRK2-PD pathogenesis was similar to that of idiopathic disease. Very recently, Jensen and colleagues (19) identified a noninclusion, aggregated α-Syn in previously considered, “non-Lewy body” LRRK2 cases. These results suggest that Lewy-body negative LRRK2-PD is not associated with a lack of α-Syn aggregation in neurons, but rather a deficiency in the appearance of inclusions.

In our prior analyses of postmortem human striatum from patients with LRRK2 mutations, idiopathic PD, or age matched controls, we detected the same cell type–specific, striatal primary cilia loss in patients with LRRK2 pathway mutations, and also in patients with idiopathic disease (12, 13). This suggested that synuclein-driven pathology might also trigger cell-type specific cilia loss. To explore this further, we sought a physiologically relevant mouse model for synuclein disease. A recently generated *Snca^G51D/G51D^* (G51D) mouse, where the mutation is knocked into the endogenous murine *Snca* locus (20) provided an ideal model. These mice express mutant α-Syn^G51D^ in the native spatiotemporal pattern and display the cascade of molecular events and symptom progression characteristic of human PD, including early loss of olfaction and enteric dysfunction followed by age-related motor symptoms (20). We show here that like LRRK2 pathway mutant animals, G51D mice show cell-type selective cilia loss and loss of striatal neuroprotection.

## Results

### Loss of Cilia in Vulnerable Striatal Interneurons and Astrocytes of G51D Mice.

**Fig. 1.**
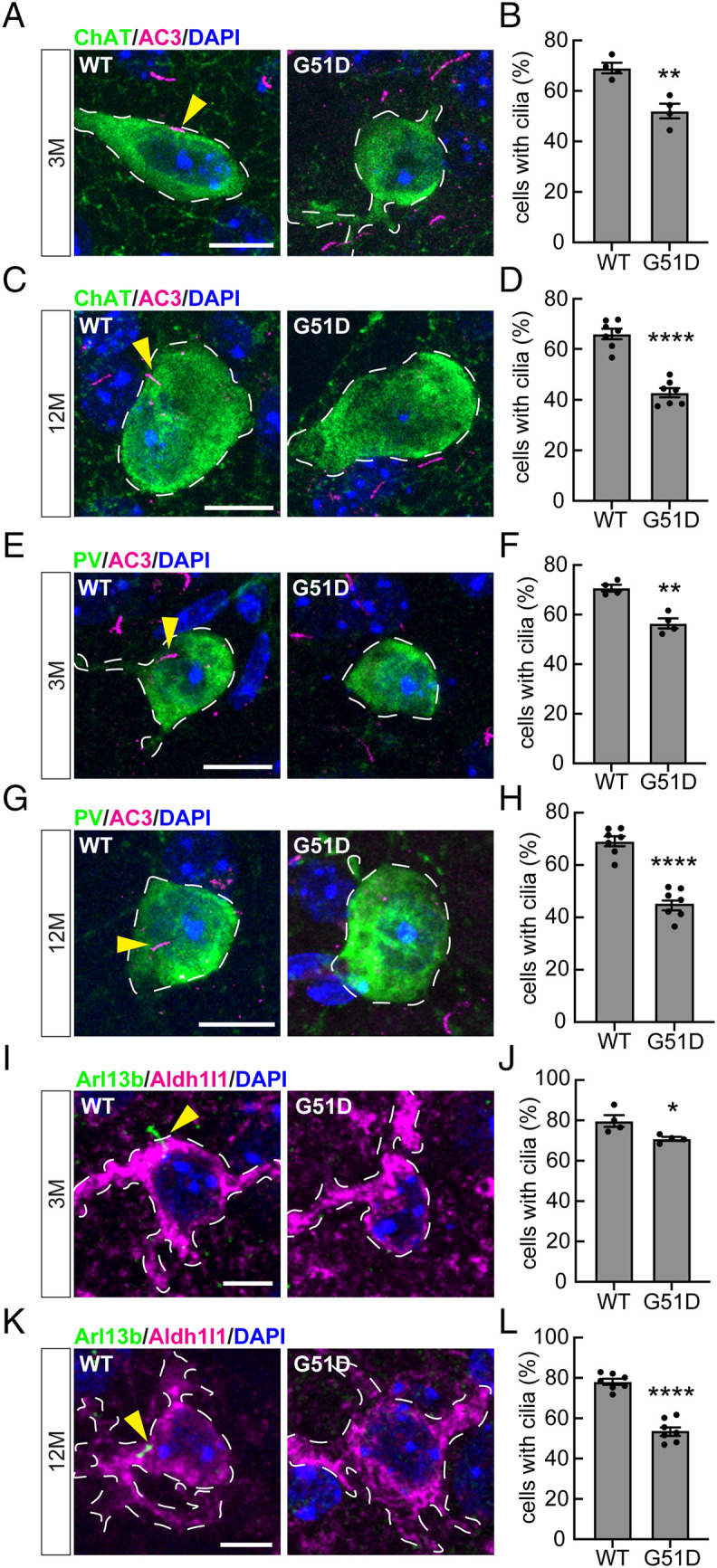
The *Snca^G51D/G51D^* mutation induces age-dependent cilia defects in vulnerable cells of the mouse dorsal striatum. (*A*, *C*, *E*, *G*, *I*, and *K*) Representative confocal immunofluorescence images of dorsal striatal sections from 3- and 12-mo-old (3 M and 12 M) wild-type and *Snca^G51D/G51D^* mice. Cholinergic and parvalbumin (PV) interneurons were labeled with anti-ChAT and anti-PV antibodies (green, as indicated), respectively in this and all subsequent figures. In all figures, neuronal primary cilia were labeled with anti-AC3 antibody (magenta; yellow arrowheads). Nuclei were labeled with DAPI (blue). Astrocytes were labeled with anti-Aldh1l1 antibody (magenta), and astrocytic primary cilia were labeled with anti-Arl13B antibody (green; yellow arrowheads). Scale bars, 10 μm for cholinergic and PV neurons; 5 μm for astrocytes. (*B* and *D*) Quantitation of ChAT^+^ neurons containing a cilium in 3- and 12-mo-old wild-type and *Snca^G51D/G51D^* mice, respectively. (*F* and *H*) Quantitation of PV neurons containing a cilium in 3- and 12-mo-old wild-type and *Snca^G51D/G51D^* mice, respectively. (*J* and *L*) Quantitation of Aldh1l1^+^ astrocytes containing a cilium in 3- and 12-mo-old wild-type and *Snca^G51D/G51D^* mice, respectively. Values represent mean ± SEM from four (3-mo-old) or seven (12-mo-old) mice per group, with two to three sections analyzed per mouse. For the 3-mo-old experiment set, 54 ± 7, 40 ± 5, and 83 ± 10 (mean ± SD) cells were counted for ChAT, PV, or ALDH1L1 cells, respectively. For the 12-mo-old experiment set, 44 ± 4, 50 ± 8, and 81 ± 12, cells were counted for ChAT, PV, or ALDH1L1 cells. Statistical significance was determined using an unpaired Student’s *t* test. **P = 0.0033 for ChAT^+^ neurons at 3 mo; ****P < 0.0001 for ChAT^+^ neurons at 12 mo; **P = 0.0012 for PV neurons at 3 mo; ****P < 0.0001 for PV neurons at 12 mo; *P = 0.0291 for Aldh1l1^+^ astrocytes at 3 mo; ****P < 0.0001 for Aldh1l1^+^ astrocytes at 12 mo.

We first examined the ciliation status of neurons in the striatum of WT and G51D mice. Fig. 1 shows examples of ChAT neurons (Fig. 1 *A*–*D*), PV neurons (Fig. 1 *E*–*H*), and ALDH1L1^+^ astrocytes (Fig. 1 *I*–*L*) and their primary ciliation status, in the dorsal striatum (*SI Appendix*, Fig. S1*A*) at 3 and 12 mo of age (3 M, 12 M). Neuronal cilia are labeled with anti-adenylate cyclase III while astrocyte cilia are visualized using anti-Arl13b antibodies. As shown in the quantitation at right, when compared with age-matched, wild-type mice, all cell types showed cilia loss even at 3 mo of age, although the astrocytes appeared to require aging before a highly significant difference was detected. Nevertheless, all cell types analyzed showed a greater loss of primary cilia with age in this G51D mouse model.

Similar to what we have previously reported for mouse genetic models and for patients with idiopathic or genetic PD, spiny projection neurons identified using anti-DARPP32 antibodies showed normal ciliation, even after 12 mo, despite expression of G51D α-Syn (*SI Appendix*, Fig. S1 *B*–*E*). These data confirm the relative resilience of the cilia of medium spiny neurons to this mutant synuclein insult.

### Loss of Cilia Impacts Hedgehog Signaling in the Dorsal Striatum.

**Fig. 2.**
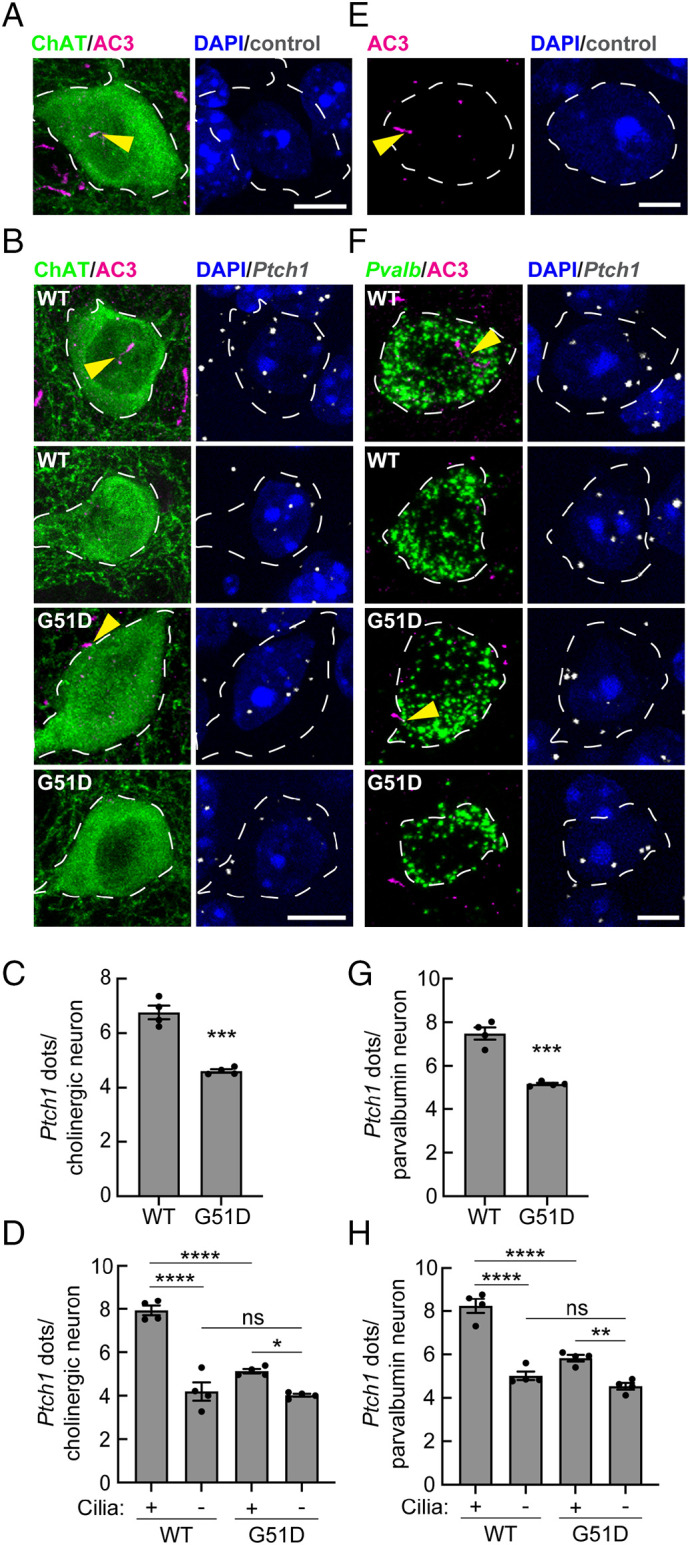
Reduced cilium-dependent PTCH1 expression in striatal cholinergic neurons of *Snca^G51D/G51D^* mice. (*A*) Control confocal immunofluorescence images of striatal cholinergic interneurons from 12-mo-old wild-type mice processed without the RNAscope probe. (Scale bar, 10 μm.) (*B*) Representative confocal immunofluorescence images of ChAT^+^ neurons from 12-mo-old wild-type and *Snca^G51D/G51D^* mice. Sections were subjected to RNAscope in situ hybridization using a *Ptch1* RNA probe (white dots). (Scale bar, 10 μm.) (*C*) Quantitation of the average number of *Ptch1* dots per ChAT^+^ neuron. (*D*) Quantitation of the average number of *Ptch1* dots segregated by ciliation status. (*E*) Control confocal immunofluorescence images of striatal parvalbumin (PV) interneurons from 12-mo-old wild-type mice processed without the RNAscope probe. Primary cilia and nuclei were labeled with anti-AC3 antibody (magenta; yellow arrowheads) and DAPI (blue), respectively. (Scale bar, 5 μm.) (*F, G* and *H*) Representative confocal immunofluorescence images of PV interneurons as in panels *B, C* and *D*. PV interneurons were identified using an RNAscope probe for *parvalbumin* (Pvalb, green). (Scale bar, 5 μm.) Values represent mean ± SEM from four mice per group, with two to three sections analyzed per mouse. More than 49 ChAT^+^ and 48 PV neurons were scored per mouse, (mean ± SD values of 53 ± 3 and 52 ± 2, respectively). Statistical significance was determined using an unpaired Student’s *t* test or one-way ANOVA with Tukey’s post hoc test. ***P = 0.0002 (ChAT^+^: wild-type versus G51D); ****P < 0.0001 (ChAT^+^: ciliated wild type versus unciliated wild type); ****P < 0.0001 (ChAT^+^: ciliated wild type versus ciliated G51D); *P = 0.0340 (ChAT^+^: ciliated G51D versus unciliated G51D). Differences were not significant (ChAT^+^: ns; *P* = 0.9577 for unciliated wild type versus unciliated G51D). ***P = 0.0002 (PV: wild type versus G51D); ****P < 0.0001 (PV: ciliated wild type versus unciliated wild type); ****P < 0.0001 (PV: ciliated wild type versus ciliated G51D); **P = 0.0061 (PV: ciliated G51D versus unciliated G51D). Differences were not significant (PV: ns; *P* = 0.4428 for unciliated wild type versus unciliated G51D).

Primary cilia are required for transducing Hedgehog signals by canonical pathways (15). We therefore predicted that loss of cilia would reduce the expression of Hedgehog target genes, such as *Patched* (Ptch1). To test this, we used RNAscope fluorescence in situ hybridization (FISH) to assess Hedgehog-dependent Ptch1 expression in ChAT and PV neurons (Fig. 2). Control reactions performed in the absence of an RNAscope probe showed no detectable RNA signal in either ChAT (Fig. 2*A*) or PV (Fig. 2*E*) neurons. Fig. 2*B* shows representative examples of ciliated and nonciliated ChAT neurons in wild-type and G51D mice at 12 mo. Quantitation revealed that G51D mice exhibited ~30% fewer Ptch1 transcripts compared to wild-type mice (Fig. 2*C*), a reduction comparable to the observed ~40% loss of cilia at this age (Fig. 1*D*). Fig. 2*D* compares the transcriptional responses of wild-type and G51D cells according to ciliation status. Wild-type ciliated cells expressed the highest level of Ptch1 RNA, and about twice as much as nonciliated wild-type cells. Even ciliated G51D cells expressed lower levels of Ptch1 RNA than ciliated wild-type ChAT neurons, suggesting that the cilia were defective in terms of their signaling capacity. Overall, loss of cilia corresponded with significant loss of Hedgehog signaling in both wild-type and G51D mutant ChAT neurons (Fig. 2*D*).

Fig. 2 *F*–*H* present a similar analysis of striatal PV neurons. Again, cilia loss resulted in a significant decrease in Ptch1 transcripts in these neurons (Fig. 2*G*). Analysis of Hedgehog signaling in ciliated versus nonciliated cells again showed a sharp decrease in Ptch1 expression in nonciliated wild-type cells and defective signaling and lower Ptch1 expression in ciliated G51D PV neurons (Fig. 2*H*). Together, these data demonstrate that Hedgehog signaling is impaired in striatal ChAT and PV neurons in the G51D mouse model; residual cilia in G51D neurons also appear to be functionally compromised.

### Loss of Striatal Neuroprotective Factors.

**Fig. 3.**
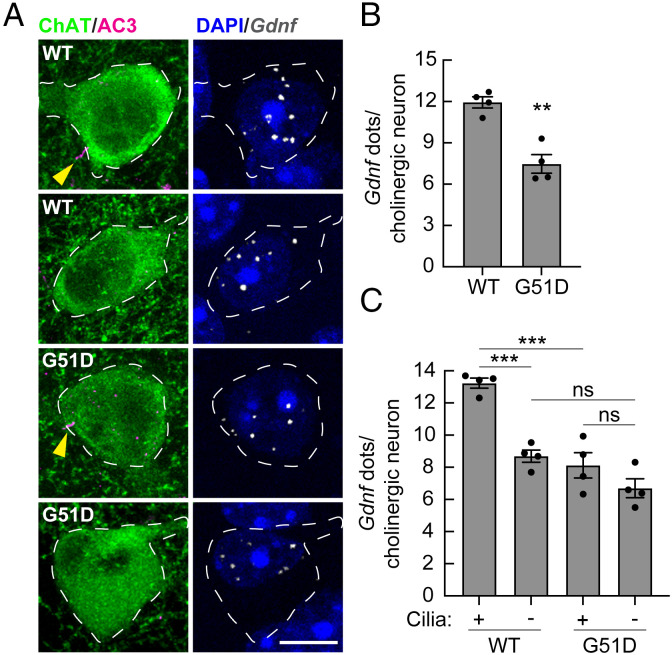
Reduced cilium-dependent GDNF expression in striatal cholinergic neurons of *Snca^G51D/G51D^* mice. (*A*) Representative confocal immunofluorescence images of cholinergic neurons from 12-mo-old wild-type and *Snca^G51D/G51D^* mice. Sections were subjected to RNAscope in situ hybridization using a *Gdnf* RNA probe (white dots). (Scale bar, 10 μm.) (*B*) Quantitation of the average number of *Gdnf* dots per ChAT^+^ cholinergic interneuron. (*C*) Quantitation of the average number of *Gdnf* dots segregated by ciliation status. Values represent mean ± SEM from four mice per group, with two to three sections analyzed per mouse. More than 49 ChAT^+^ neurons were scored per mouse, corresponding to a mean ± SD of 59 ± 6. Statistical significance was determined using an unpaired Student’s *t* test or one-way ANOVA with Tukey’s post hoc test. **P = 0.0013 (wild type versus G51D); ***P = 0.0004 (ciliated wild type versus unciliated wild type); ***P = 0.0001 (ciliated wild type versus ciliated G51D). Differences were not significant (ns; *P* = 0.0983 for unciliated wild-type versus unciliated G51D; *P* = 0.3053 for ciliated G51D versus unciliated G51D).

**Fig. 4.**
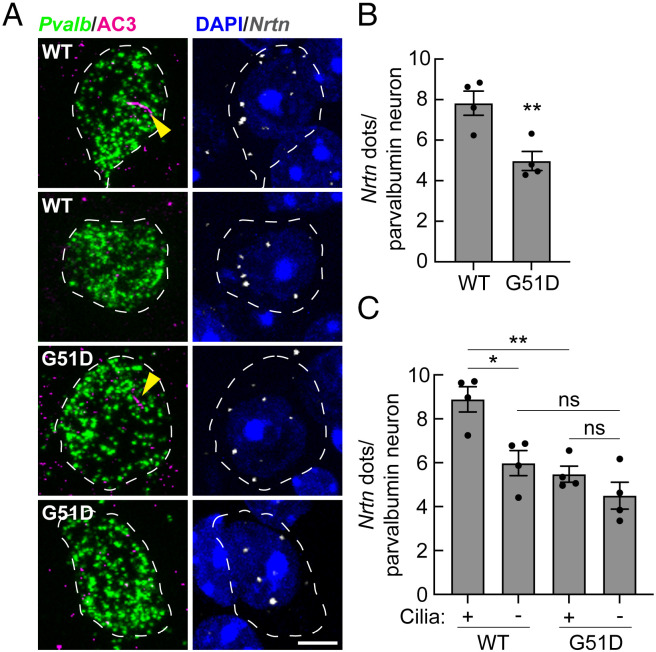
Reduced cilium-dependent Neurturin expression in striatal parvalbumin neurons of *Snca^G51D/G51D^* mice. (*A*) Confocal immunofluorescence images of parvalbumin (PV) neurons from 12-mo-old wild-type and *Snca^G51D/G51D^* mice. PV interneurons were identified as in Fig. 2 (green). Sections were subjected to RNAscope in situ hybridization using a *Nrtn* RNA probe (white dots). (Scale bar, 5 μm.) (*B*) Quantitation of the average number of *Nrtn* dots per PV interneuron. (*C*) Quantitation of the average number of *Nrtn* dots segregated by ciliation status. Values represent mean ± SEM from four mice per group, with two to three sections analyzed per mouse. More than 49 PV neurons were scored per mouse, corresponding to a mean ± SD of 55 ± 7. Statistical significance was determined using an unpaired Student’s *t* test or one-way ANOVA with Tukey’s post hoc test. **P = 0.0091 (wild type versus G51D); *P = 0.0115 (ciliated wild type versus unciliated wild type); **P = 0.0037 (ciliated wild type versus ciliated G51D). Differences were not significant (ns; *P* = 0.2625 for unciliated wild type versus unciliated G51D; *P* = 0.5898 for ciliated G51D versus unciliated G51D).

Loss of cilia would be predicted to lead to loss of transcription of cilia- and Hedgehog-dependent neurotrophic factors (15). We used RNAscope FISH to monitor expression of the neurotrophic factors, GDNF (Fig. 3), NRTN (Fig. 4), and BDNF (*SI Appendix*, Fig. S2) in specific cell types in the dorsal striatum. Fig. 3 shows examples of ciliated and nonciliated ChAT neurons in wild-type and G51D mice. Quantitation of these data revealed that G51D mice express fewer GDNF transcripts than wild-type mice, with the decrease proportional to their cilia loss at 12 mo of age, ~40% (Figs. 1*D* and 3*B*). Moreover, loss of GDNF expression was cilia-dependent (Fig. 3*C*). Fig. 4 shows similar analysis of PV neurons (Fig. 4 *A* and *B*); again, cilia loss led to significant loss of NRTN transcripts in striatal PV neurons (Fig. 4*B*) and expression was cilia dependent (Fig. 4*C*). Overall, both GDNF and NRTN expression correlated with primary ciliation in WT cells; however, even ciliated G51D cells showed reduced production, consistent with impaired functionality of the remaining cilia. Finally, analysis of ALDH1L1^+^ astrocytes in this brain region revealed a marked reduction in BDNF production (*SI Appendix*, Fig. S2 *A* and *B*). Although cilia dependence was less apparent in the present dataset (*SI Appendix*, Fig. S2*C*), our prior studies in 5-mo mice showed that BDNF is a cilia-dependent gene (17), supporting the interpretation that astrocytic BDNF reduction reflects disrupted cilia-associated signaling.

### Loss of Primary Cilia in the Piriform Cortex.

**Fig. 5.**
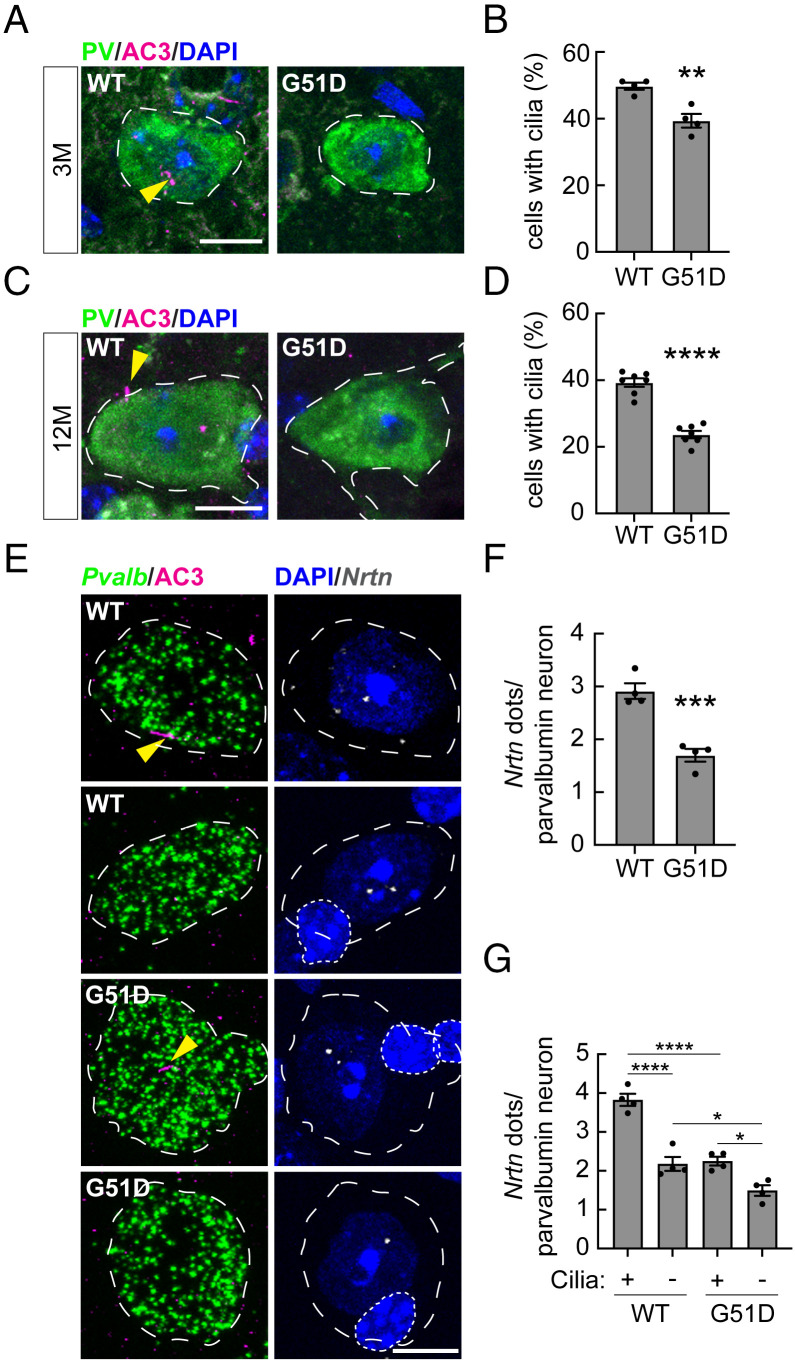
The *Snca^G51D/G51D^* mutation induces age-dependent cilia defects and reduces Neurturin expression in parvalbumin neurons of the piriform cortex. (*A* and *C*) Confocal immunofluorescence images of parvalbumin (PV) neurons from 3- and 12-mo-old wild-type and *Snca^G51D/G51D^* mice. PV interneurons were labeled as in Fig. 1 (green). (Scale bars, 10 μm.) (*B* and *D*) Quantitation of PV neurons containing a cilium in 3- and 12-mo-old wild-type and *Snca^G51D/G51D^* mice, respectively. (*E*) Confocal images of PV neurons from 12-mo-old wild-type and *Snca^G51D/G51D^* mice. Sections were subjected to RNAscope in situ hybridization using a *Nrtn* and *Pvalb* RNA probe (white or green dots). The longer dashed line outlines the soma of a PV neuron whereas the shorter dashed line marks adjacent cell(s) intersected by the longer dashed line. (Scale bars, 10 μm.) (*F*) Quantitation of the average number of *Nrtn* dots per PV interneuron. (*G*) Quantitation of the average number of *Nrtn* dots segregated by ciliation status. Values represent mean ± SEM from four mice per group, except for Fig. 5*D*, which includes seven (12-mo-old) mice for the ciliation analysis, with two to three sections analyzed per mouse. For the ciliation experiments, in both the 3-mo-old and 12-mo-old groups, 49 ± 5 and 49 ± 4 (mean ± SD) respectively, were counted. For the RNAscope experiment, 62 ± 4 cells were counted. Statistical significance was determined using an unpaired Student’s *t* test or one-way ANOVA with Tukey’s post hoc test. **P = 0.0040 for PV neurons at 3 mo; ****P < 0.0001 for PV neurons at 12 mo; ***P = 0.0007 (wild type versus G51D); ****P < 0.0001 (ciliated wild type versus unciliated wild type; ciliated wild type versus ciliated G51D); *P = 0.0285 (unciliated wild type versus unciliated G51D); *P = 0.0155 (ciliated G51D versus unciliated G51D).

An important prodromal symptom of PD is loss of olfaction (21). G51D mice show phosphoSer129 (pS129) α-Syn accumulation in the piriform cortex that likely contributes to their olfactory deficits (20). PV interneurons were selected for analysis because they provide key local inhibition in the piriform cortex and are essential for shaping odor responses, including odor processing and learning in this region. Moreover, our prior observation of cilia loss in PV neurons in the dorsal striatum suggested that this interneuron population may be particularly vulnerable in this model (22–24). In the piriform cortex of wild-type, 3-mo-old mice, PV neurons were 50% ciliated (Fig. 5 *A* and *B*) compared with 70% ciliated in the dorsal striatum at this age (Fig. 1*F*). At 12 mo, these values decreased to 40% ciliated in the wild-type piriform cortex (Fig. 5 *C* and *D*) compared with 70% in the striatum (Fig. 1*H*). Thus, even in wild-type mice, cilia are more likely to be lost with age in this brain region.

In G51D mice, as early as 3 mo, but more significantly at 12 mo, PV neurons lost almost half of their primary cilia, on top of the loss due to aging (Fig. 5 *A*–*D*). At 6 mo, G51D mice show olfactory deficits (20), so one might have predicted that cilia loss would have been greater in PV neurons in this brain region compared with the striatum. NRTN expression in PV neurons is significantly decreased by 12 mo, in direct proportion to ciliary loss (Fig. 5 *E* and *F*). We detected a ~50% reduction in NRTN transcripts in G51D mice, and both ciliated and nonciliated piriform cortex PV neurons showed reduced NRTN RNA relative to controls. Again, NRTN RNA production was cilia-dependent, similar to that in the striatum (Fig. 4*C*), and as we have previously reported in other genetic backgrounds (Fig. 5*G*; 13, 14). Absolute NRTN transcript levels were lower in wild-type piriform cortex compared with wild-type striatum, measured in the same tissue sections (~8 versus ~3 dots per cell) but the signal was reproducible across sections and sufficient for quantitative comparison. The lower NRTN abundance in piriform cortex PV neurons may reflect the lower baseline ciliation rate of PV neurons in this region. Beyond general neuroprotection, the full consequences of PV neuron cilia loss in both piriform cortex and striatum remain to be established.

### Analysis of Cilia Loss in the Olfactory Epithelium.

Peripheral olfactory processing begins in the olfactory epithelium, where olfactory sensory neurons (OSNs) detect odorants and project their axons to the olfactory bulb (25, 26). OSNs are multiciliated neurons in which olfactory G protein–coupled odorant receptors are localized to the sensory cilia (27, 28). Each OSN expresses just one out of ~400 different odorant receptors, and all sensory neurons expressing a given receptor project to a unique pair of glomeruli in the olfactory bulb (28, 29). Other ciliated cells in the olfactory epithelium are the horizontally oriented basal cells, which are singly ciliated, quiescent reserve stem cells that become activated after injury to regenerate both OSNs and non-neuronal epithelial cell types (30). Joiner et al. (31) demonstrated that horizontal basal cells require their primary cilia to support regeneration of the olfactory epithelium following injury.

**Fig. 6.**
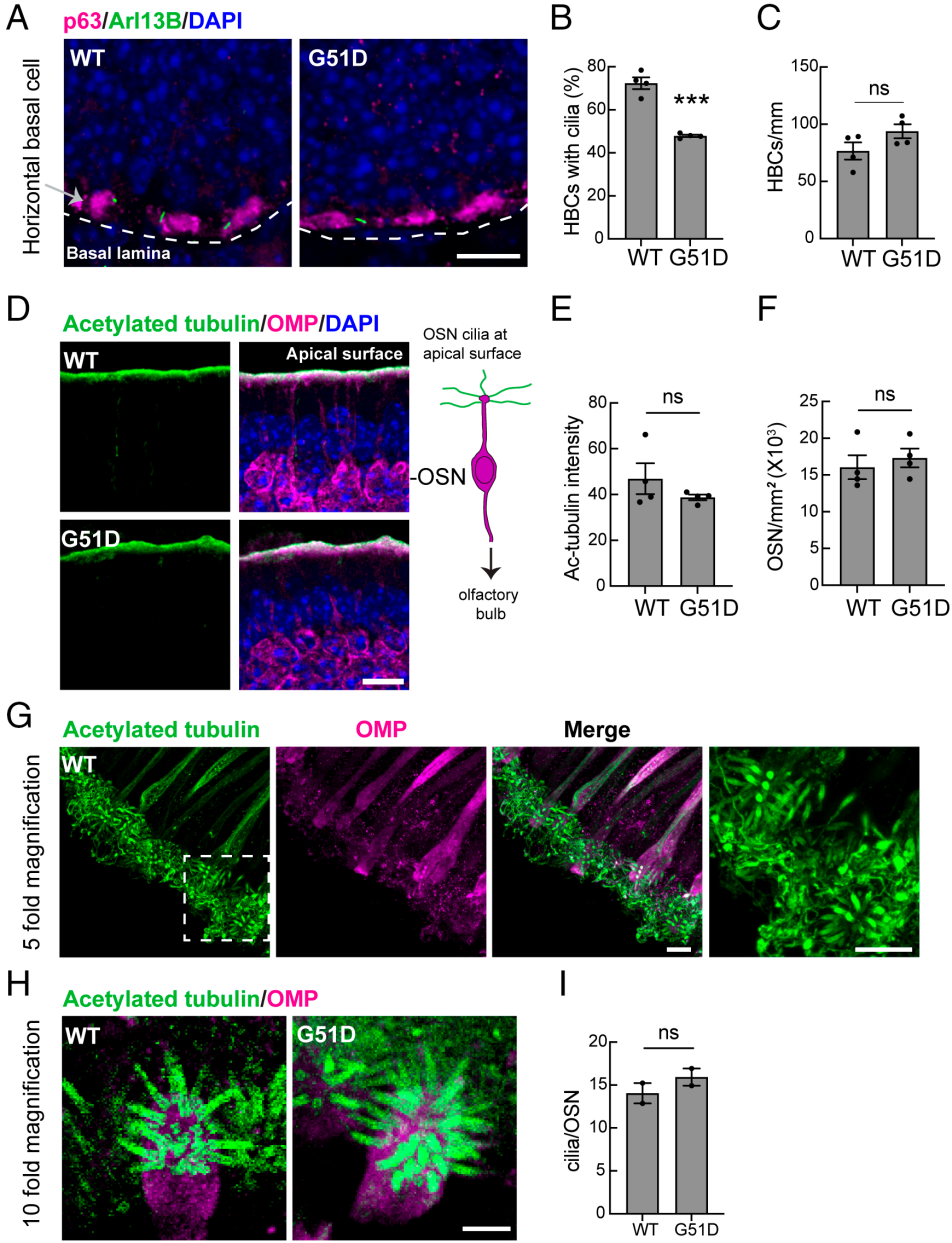
Primary cilia loss in the olfactory stem cells of 12-mo *Snca^G51D/G51D^* mice. (*A*) Representative images of horizontal basal cells (HBCs) from the olfactory epithelium of 12-mo-old wild-type and *Snca^G51D/G51D^* mice. HBCs were labeled with anti-p63 antibody (magenta), primary cilia with anti-Arl13B antibody (green), and nuclei with DAPI (blue). Dashed lines indicate the basement membrane. (Scale bar, 10 µm.) (*B*) Quantification of the percentage of HBCs containing a cilium. 125 ± 23 HBCs were scored per mouse from 2 to 3 sections. (*C*) Quantification of HBCs per mm of the olfactory epithelium. More than 25 fields were analyzed per animal, corresponding to mean ± SD values of 27 ± 2. (*D*) Representative images of the olfactory epithelium of 12-mo- wild-type and *Snca^G51D/G51D^* mice. Olfactory sensory neurons (OSNs) in *D*, *G*, and *H* were labeled with anti-OMP antibody (magenta), cilia with anti-Acetylated alpha-tubulin (green), and nuclei with DAPI (blue). (Scale bar, 10 µm.) (*E*) Quantitation of intensity of acetylated tubulin at the apical surface of the olfactory epithelium. 15 fields were analyzed per animal, corresponding to mean ± SD values of 15 ± 0. (*F*) Quantitation of OSNs. 15 fields were analyzed per animal, corresponding to mean ± SD values of 15 ± 0. (*G*) Representative confocal images of the OSN cilia of 12-mo-old wild-type mice visualized using expansion microscopy. (Scale bar, 2 µm.) (*H*) Representative confocal images of the OSN cilia of 12-mo-old wild-type and *Snca^G51D/G51D^* mice visualized using expansion microscopy. (Scale bar, 1 µm.) (*I*) Quantitation of cilia per OSN. More than 15 OSNs were scored per animal, corresponding to mean ± SD values of 17 ± 3. Values represent mean ± SEM from four mice per group for Fig. 6 *B*, *C*, *E*, and *F*, and from two mice per group for Fig. 6*I*. Statistical significance was determined using an unpaired Student’s *t* test. ***P = 0.0001 for WT versus *Snca^G51D/G51D^* mice.

To gain further insight into the loss of olfaction in G51D mice, we also explored the ciliation status of their olfactory epithelial cells. Fig. 6*A* shows staining of horizontal basal cells, labeled with anti-p63 and their single, primary cilia (labeled with anti-Arl13B), in wild-type and G51D α-Syn olfactory epithelium. Quantification of ciliation status and cell number showed ~30% loss of cilia (Fig. 6*B*) with no significant loss of cell number (Fig. 6*C*). Cilia loss will decrease the ability of these cells to respond to Hedgehog and other growth and differentiation signals needed during regeneration processes (31).

OSNs were identified using anti-olfactory marker protein (OMP) antibodies; apically localized cilia were labeled with anti-acetylated tubulin (Fig. 6*D*). Ciliary staining was restricted to the apical surface of the olfactory epithelium, but individual cilia could not be resolved by conventional fluorescence microscopy (Fig. 6*D*). Nevertheless, the intensity of staining was not different in these micrographs between wild-type and G51D mice (Fig. 6*E*) and the overall number of OSNs was also unchanged (Fig. 6*F*). To increase the resolution of our analyses, we incorporated 4× expansion microscopy and Airyscan superresolution to enable visualization of entire, multiciliated OSN tufts (Fig. 6 *G* and *H* and *SI Appendix*, Fig. S3). Although it was sometimes complicated to count, precisely, the number of individual cilia within each multiciliated tuft, we did not discern any obvious differences between the multiciliation status of OSNs, when comparing wild-type and G51D α-Syn olfactory epithelium (Fig. 6*I*). Note that the molecular mechanisms that generate multiple cilia on a single OSN differ from those that underlie formation of the more common single primary cilium (32–34), so it is perhaps not unexpected that these cell types respond differently to the G51D mutation. Altogether, these data suggest that olfactory regeneration may be impacted by loss of cilia on horizontal basal cells; further experiments will be needed to pinpoint more precisely, the full defects underlying olfactory dysfunction in this synucleinopathy model.

### Overall LRRK2 Pathway Activation Appears Unchanged.

To consolidate an enormous amount of work over many years, we have created a heat map chart (*SI Appendix*, Fig. S4*A*) that summarizes cilia vulnerability in distinct cell types in various brain regions that we have analyzed to date. Cilia loss in 12-mo-old G51D mice was comparable to that seen for ChAT neurons in the striatum of 13-mo-old LRRK2 G2019S mice and somewhat less than that seen in LRRK2 R1441C mice at 7 mo of age, the most impacted example (*SI Appendix*, Fig. S4*A*). We will continue to update this chart as additional data become available.

Because LRRK2 activation and Rab GTPase phosphorylation are known to block primary cilia formation (8, 35, 36), one possible explanation for cilia loss in the G51D mouse model is that α-Syn aggregates cause lysosome stress, thereby activating LRRK2 kinase (37–39). To test this, we harvested brains from 9-mo-old mice and analyzed the tissue by western blot (*SI Appendix*, Fig. S4 *B* and *C*). We failed to detect significant differences in levels of pRab10, pRab12, or LRRK2 kinase when comparing wild-type and G51D mice at the level of whole brain tissue. It is possible that LRRK2 is only activated in select cell types that are vulnerable to cilia loss, in which case such activation would not be able to be detected in a whole brain lysate. Indeed, even in a mouse where LRRK2 is inappropriately activated in all cell types, increases in pRab12 are ~25% in overall magnitude in whole brain tissue (cf. ref. 10).

### PhosphoSer129 Synuclein Levels Correlate with Cilia Loss in Vulnerable Cells.

**Fig. 7.**
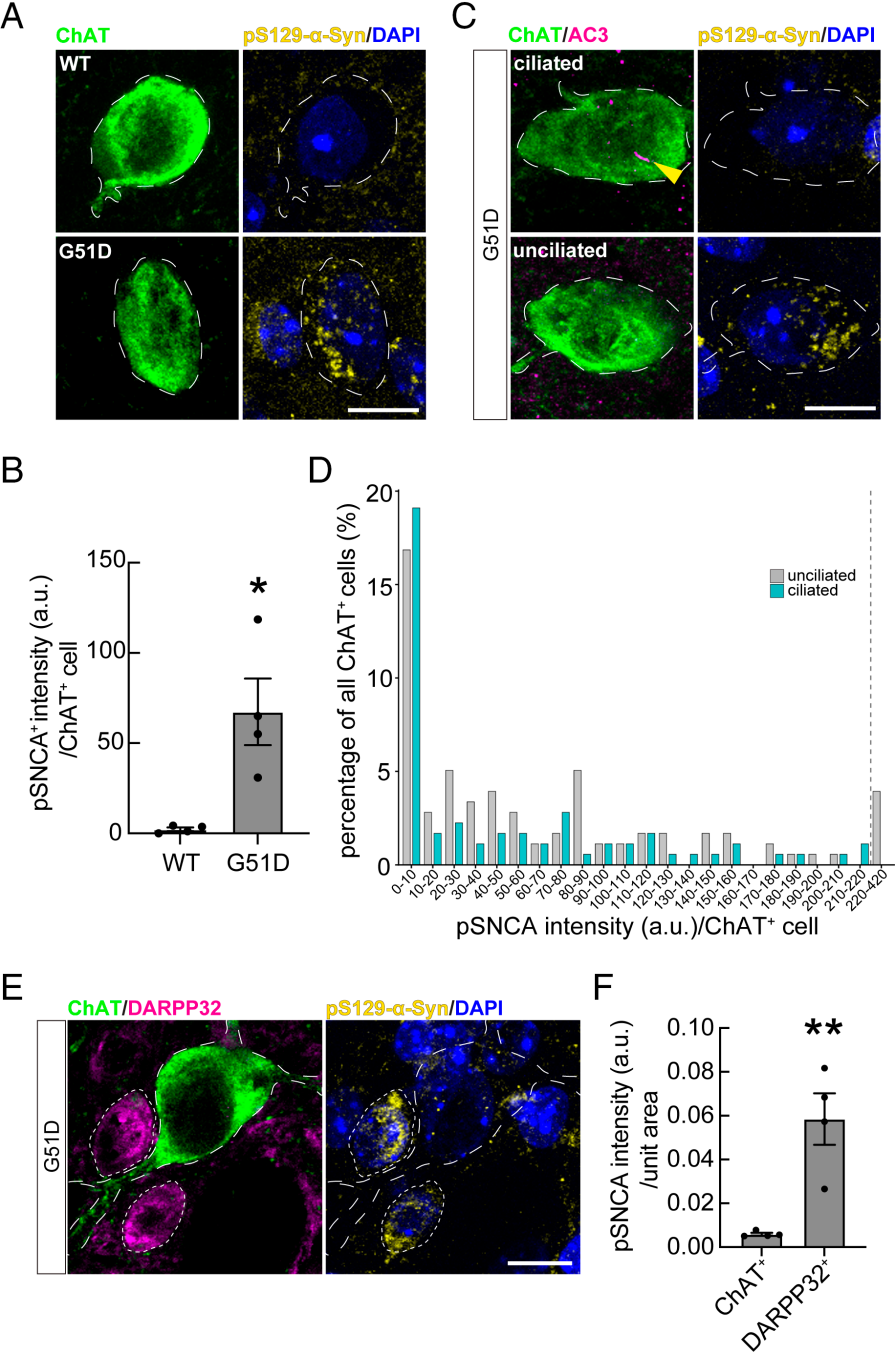
Loss of primary cilia correlates with increased phosphorylated α-synuclein (pSNCA) accumulation in striatal cholinergic neurons of *Snca^G51D/G51D^* mice. (*A*) Confocal immunofluorescence images of cholinergic neurons from 12-mo-old wild-type and *Snca^G51D/G51D^* mice. In all panels, phosphorylated α-synuclein (pSNCA) was labeled with anti-pSNCA (Ser129) antibody (yellow). (Scale bars, 10 μm.) (*B*) Quantification of pSNCA^+^ intensity (arbitrary units) per ChAT^+^ interneuron. (*C*) Confocal images of ChAT neurons from 12-mo *Snca^G51D/G51D^* mice. (Scale bars, 10 μm.) (*D*) Single cell correlation between ciliation status and pSNCA intensity in ChAT^+^ neurons from *Snca^G51D/G51D^* mice. Single-cell pSNCA intensity was quantified per ChAT^+^ interneuron and binned, with cells plotted as the percentage of all ChAT^+^ neurons within each intensity range. Data are stratified by cilia status (ciliated, green; unciliated, gray). The dashed vertical line indicates a transition between fine and coarse binning. The analyzed population is the same as in Fig. 7*B*. (*E*) Confocal images of cholinergic and medium spiny neurons from 12-mo *Snca^G51D/G51D^* mice. Cholinergic interneurons are circled (long dashed outlines); medium spiny neurons (MSNs) were labeled with anti-DARPP32 antibody (magenta, short-dashed outlines). (Scale bars, 10 μm.) (*F*) Quantification of the pSNCA^+^ intensity (arbitrary units, a.u.) per ChAT^+^ or DARPP32^+^ unit area from 12-mo *Snca^G51D/G51D^* mouse brains. Values represent mean ± SEM from four mice per group (Fig. 7*B*) or per mouse per cell type (Fig. 7*F*). For Fig. 7*B*, two to three brain sections were analyzed per mouse, with 50 ChAT^+^ neurons scored per mouse, corresponding to mean ± SD values of 50 ± 0. For Fig. 7*F*, neurons were quantified across 45 serial image sections and averaged to obtain one value per brain, corresponding to a mean ± SD value of 45 ± 0. Statistical significance was determined using an unpaired Student’s *t* test. *P = 0.0126; **P = 0.0043.

Phosphorylation of α-synuclein at Serine 129 (pS129-α-Syn) is a major posttranslational modification seen in postmortem human PD tissue (40). In G51D mice, pS129-α-Syn is detected in the motor cortex and structures necessary for motor coordination, including the striatum, pons, brainstem, and spinal cord (20). We evaluated pS129-α-Syn levels in the striatum, comparing ChAT neurons that are vulnerable to cilia loss with spiny projection neurons that are not. G51D mice showed much higher levels of pS129-α-Syn in ChAT neurons compared with wild-type mice (Fig. 7 *A* and *B*).

Quantification of pS129-α-Syn relative to ciliation status showed that, among striatal ChAT neurons, those with higher pS129-α-Syn levels were less likely to be ciliated (Fig. 7 *C* and *D*). Panel *D* shows how pS129–α-Syn immunoreactivity is distributed among G51D striatal ChAT neurons, and how ciliation correlates across that range of staining intensities. For each 10-unit bin of pS129–α-Syn intensity (X-axis), the bars indicate the percentage of ChAT neurons that fall into that bin and whether those neurons are ciliated (teal) or unciliated (gray). In the lowest α-Syn bin (0 to 10 a.u.), the proportion of ciliated ChAT neurons matches the overall ciliation rate in G51D ChAT cells (about 45%). As pS129–α-Syn intensity increases beyond this range, the proportion of unciliated cells increases, indicating that ChAT neurons with higher pS129–α-Syn accumulation are disproportionately unciliated. Thus, elevated pS129–α-Syn in ChAT neurons is associated with reduced likelihood of primary cilium presence.

In contrast, neighboring spiny projection neurons, which are far more abundant, retain their primary cilia in mouse and human PD models, including G51D (*SI Appendix*, Fig. S1 *B*–*E*), yet exhibit much higher pS129-α-Syn levels than ChAT neurons in the same striatal region (Fig. 7 *E* and *F*). Notably, at 12 mo, the striatum overall has lower pS129–α-synuclein burden than other brain regions (20). Thus, pS129-α-Syn levels correlate with cilia loss within ChAT neurons but not within spiny projection neurons. This mirrors our previous finding that spiny projection neurons express more LRRK2 than ChAT neurons, yet ChAT neurons are more susceptible to cilia loss in mice expressing hyperactive, pathogenic LRRK2. Together, these data indicate that pS129-α-Syn levels do not predict overall vulnerability to cilia loss across different neuronal cell types.

## Discussion

G51D α-Syn mice provide a valuable model of idiopathic human PD, showing early olfactory deficits and age-dependent loss of motor function (20). We have shown here that G51D mice show cellular phenotypes shared by multiple genetic models of PD including mice and humans with pathogenic *LRRK2* mutations (8, 12–14, 35) or loss of the LRRK2-reversing PPM1H or PPM1M phosphatases (9, 10) and PINK1 knockout mice (41). These phenotypes include loss of primary cilia on cholinergic and parvalbumin neurons and ALDH1L1^+^ astrocytes (but not on the more abundant spiny projection neurons) in the dorsal striatum that are important for Hedgehog-dependent neurotrophic factor production in support of dopamine neurons. Although mouse models do not fully recapitulate human PD phenotypes such as significant loss of dopamine neurons in the substantia nigra and resting tremor, the G51D model has advantages in that it involves α-Syn expression from the endogenous mouse locus and provides spatiotemporal expression of mutant α-Syn that is analogous to PD. Indeed, despite increasing levels of pS129-α-Syn in the substantia nigra at 12 and 18 mo of age, and primary cilia and neurotrophic factor loss at earlier stages, these mice nevertheless show mild loss of dopamine neurons. The motor phenotypes seen in G51D mice at 9 to 12 mo of age may be due to a diminished dopaminergic axon arbor in the striatum rather than absolute nigral cell loss.

G51D mice show initial deposition of α-Syn phosphorylation in the olfactory tract and vagal and enteric nerves, later becoming detectable along the olfactory circuit to the piriform cortex, entorhinal cortex, and hippocampus, and from the vagus nerve to the substantia nigra (20). The mice show delayed increases in pS129-α-Syn, which is not detectable at 3 mo of age outside of the olfactory system and only becomes apparent at 12 mo of age in deeper layers of the cortex, hippocampus, and substantia nigra (20). Mice display motor deficits from 9 mo of age but do not show a significant reduction in dopamine levels until 18 mo of age. These findings support the contention that early motor deficits are driven by dopaminergic neuronal dysfunction rather than absolute neuronal loss (42). In the striatum at 12 mo of age, we detected increased pS129-α-Syn in G51D but not in wild-type mice, with much more pronounced expression in spiny projection neurons than in ChAT neurons. Despite this distribution, ChAT neurons selectively lost primary cilia while the spiny projection neurons did not. Thus, important physiological changes in the striatum are unlikely to correlate directly with the overall pS129-α-Syn burden observed in this study. [Note that the images presented here are at high magnification and intensities cannot be compared directly with lower magnification images presented previously (20)]. In addition, a limitation of this study is that ciliation was scored in absolute terms, albeit from brains harvested and fixed at the same time of day. Primary cilia can respond dynamically to signaling disturbances via morphological changes tied to physiological state shifts. Future studies will be needed to explore whether stress or translation changes precede ciliopathy in vulnerable neurons.

Unlike what we have seen in LRRK2 mutant mice where GFAP^+^ astrocytes display a major loss of cilia as young as 10 wk of age (8), G51D astrocytes appeared to require aging before a highly significant astrocytic ciliation difference was detected. Since astrocytes are filled with lysosomes, it is possible that this result can be explained by astrocytic capacity to degrade α-Syn aggregates (43, 44), explaining their relative decreased vulnerability to G51D α-Syn expression.

As discussed earlier, one of the first nonmotor PD symptoms is loss of olfaction (21, 45). Odorants are first detected by peripheral olfactory sensory neurons of the olfactory epithelium. These neurons rely on their cilia for GPCR-mediated odorant detection (28) and given the impact of multiple PD-linked mutations on ciliary signaling, we were excited to explore OSN ciliary status in the G51D mice. We incorporated a protocol for isotropic expansion of the olfactory epithelium to enable superresolution characterization of these multiciliated neurons. Under conditions of highly improved microscopic resolution, the cilia appeared fully intact on G51D olfactory sensory neurons. As mentioned earlier, the pathway to multiciliation is different from that used by cells containing a single primary cilium (33, 34, 46); nevertheless, it was important to evaluate olfactory sensory neuron ciliary status. In contrast, we did detect loss of primary cilia on horizontal basal cells of the olfactory epithelium that require cilia to enable them to regenerate damaged olfactory epithelia (31).

Upstream in the olfactory pathway, the piriform cortex integrates olfactory bulb inputs and forms widespread connections with other brain regions (22, 23). PV neurons within the piriform cortex regulate olfactory processing by sharpening sensory encoding and enhancing odor discrimination (23, 24), and their excitability is increased by dopamine (47). Functional MRI studies show decreased activation and weaker integration with the broader olfactory–network during odor stimulation in PD compared with healthy controls (48), implicating the piriform circuits as a key locus of PD-related olfactory disruption. We observed ciliary loss and reduced NRTN production in piriform PV neurons, suggesting that cilia loss or dysfunction may impair PV neuron activity, local interneuron maintenance, or survival. Future work will reveal the full consequences of piriform PV neuron cilia loss and corresponding decrease in NRTN production.

Idiopathic PD is thought to be driven by α-Syn pathology (49). We previously reported cell-type specific cilia loss in postmortem striatal tissue from patients with idiopathic PD (12, 13), consistent with the proposal that cell type–specific ciliation is influenced by α-Syn pathology. Our findings with G51D mice support this conclusion. How might mutant α-Syn trigger cilia loss? One possible explanation would be that α-Syn aggregates activate LRRK2, a kinase that regulates ciliogenesis and is activated by lysosome stress (37, 50). It will be important to evaluate the phenotypes of LRRK2^−/−^ or LRRK2-inhibited G51D α-Syn mice, to test this hypothesis in future experiments. Prior work showed that hyperactive LRRK2 or LRRK2 overexpression exacerbated synuclein pathology (51, 52) but striatal and piriform cortex ciliation was not evaluated. Independent of LRRK2, ciliation is linked to induction of autophagy (53, 54); α-Syn aggregates might overload the autophagy machinery (55) and in that manner, somehow disrupt ciliogenesis. Future work will be needed to explain the molecular basis of LRRK2-dependent or independent ciliogenesis blockades and the resistance of medium spiny neurons to cilia loss in both mouse and human.

In addition to controlling primary ciliogenesis, LRRK2 catalyzes lysosome exocytosis, especially in cells of the macrophage lineage (56–58). While exocytic release of lysosomal α-Syn aggregates may be beneficial for neurons, microglial uptake of these aggregates and their subsequent (LRRK2-stimulated) exocytosis may also contribute to unfavorable α-Syn spread. Thus, LRRK2 may protect neurons in the short term but contribute to disease pathology in the longer term. Future experiments with the G51D α-Syn mouse model should be able to provide important information related to whether LRRK2 inhibition is beneficial or instead, exacerbates α-Syn pathology in the brain. Generation of mice with cell type–specific expression of LRRK2 may also help determine the cells in which it plays the most significant role, be it a subset of neurons, astrocytes, microglia, or all three.

Hedgehog signaling is critical for dopamine neuron survival (16) and 3 mo of LRRK2 inhibitor feeding can restore primary cilia, Hedgehog signaling and downstream, striatal neuroprotective factor production in *Lrrk2* mutant mice (14). These findings hold great promise for patients with LRRK2 mutations and encourage therapeutic development of this class of small molecule LRRK2 inhibitors. It will be very important to understand the contribution of LRRK2 hyperactivity to idiopathic PD pathophysiology to determine whether LRRK2 inhibitors will be beneficial to the larger number of patients with idiopathic PD.

In summary, we have found mice expressing G51D α-Syn share vulnerability to cilia loss in the striatum with mice carrying pathogenic LRRK2 mutations or with PPM1H, PPM1M, or PINK1 gene knockouts: Striatal cholinergic and parvalbumin interneurons, as well as astrocytes, lose primary cilia and the neurotrophic signaling needed to sustain dopaminergic neurons. We showed that these vulnerable cells do not contain higher levels of pS129-α-Syn than the surrounding spiny projection neurons that contain significantly more. We showed previously that striatal spiny projection neurons express more LRRK2 RNA than the vulnerable neurons, but in a given class of neuron, those with higher LRRK2 showed increased probability of cilia loss (12). Despite sharing a similar phenotype, the timing of cilia loss differed among these models. For example, astrocyte cilia loss was only truly notable at 12 mo in G51D mice while it is readily observed in LRRK2 pathway mutant mice at 10 wk of age. This suggests that LRRK2 is a more direct regulator of ciliogenesis than α-Syn aggregates. Yet the ages of onset for LRRK2 carriers are not so different from that of patients with idiopathic disease. Perhaps this is because apparently earlier-acting LRRK2 mutations impact about half of cholinergic and parvalbumin interneurons at all ages, while later acting α-Syn may more slowly, eventually, impact all. Altogether, these experiments highlight the importance of Hedgehog signaling in multiple brain regions for neuroprotection, especially for dopamine neuron survival. And primary cilia must also play other fundamental roles in neuronal regulation, as they receive axonal inputs from diverse neuronal types (59, 60).

## Materials and Methods

### Research Standards for Animal Studies.

Mice were housed in a level 3, American Association for Laboratory Animal Science–certified facility on a 14-h light cycle. Husbandry, housing, humane killing of animals, and experimental guidelines were approved by the Institutional Animal Care & Use Committee (IACUC) at Baylor College of Medicine. The *Snca^G51D/G51D^* knock-in mice were generated at Baylor College of Medicine; genotyping was confirmed by genomic PCR with different primers between the unmodified wild-type and modified G51D alleles (20).

### Mouse Brain and Olfactory Epithelium Processing.

*Snca^G51D/G51D^* mouse brains (3- and 12-mo-old) and age-matched wild-type controls were fixed by transcardial perfusion using 4% paraformaldehyde (PFA) in PBS as described 10.17504/protocols.io.bnwimfce. Following perfusion, whole brains were extracted, and the olfactory epithelium was rapidly dissected as described (61). Both brains and olfactory epithelium were postfixed in 4% PFA for 24 h and then immersed in 30% (w/v) sucrose in PBS until the tissue settled to the bottom of the tube (~48 h). The brains and olfactory epithelium were harvested at Baylor College of Medicine and sent to Stanford University for analysis. Prior to cryosectioning, tissues were embedded in cubed-shaped plastic blocks with OCT (BioTek, USA) and stored at −80 °C. OCT blocks were allowed to reach −20 °C for ease of sectioning. The brains and olfactory epithelium were oriented to cut coronal sections on a cryotome (Leica CM3050S, Germany) at 20 μm thickness for brain tissue and 14 µm thickness for olfactory epithelium and positioned onto SuperFrost plus tissue slides (Thermo Fisher, USA).

### Mouse Tissue Homogenization and Immunoblotting Analysis.

*Snca^G51D/G51D^* (9-mo-old) and age-matched wild-type controls were euthanized, and whole brains were harvested and immediately frozen on dry ice. Frozen brain tissues were collected at Baylor and shipped on dry ice to Stanford for downstream analysis. Frozen brain tissues were homogenized as described in 10.17504/protocols.io.sqgedtw. Briefly, frozen brain tissues were homogenized using M tubes in a GentleMACS machine with lysis buffer (50 mM Tris–HCl pH 7.4, 150 mM NaCl, 1 mM EGTA, 2% glycerol, cOmplete EDTA-free protease inhibitor cocktail (Roche), PhosSTOP phosphatase inhibitor cocktail (Roche), 1 μg/mL microcystin-LR (Sigma), and 1% (v/v) Triton X-100). 6 mL of lysis buffer was used per brain, about 500 to 600 mg frozen weight each. Tissues were homogenized using the protein cycle, run twice with 5 min incubation on ice between each cycle. Following homogenization, M-tubes were spun briefly at 2,000 × g at 4 °C for 5 min to pellet any unhomogenized material. The supernatant was divided into 1.5 mL Eppendorf tubes, then further clarified by centrifugation at 20,000 × g at 4 °C for 30 min.

See Key Resource Table (*SI Appendix*, Table S1) for primary and secondary antibodies used and their dilutions. Primary antibodies were diluted in 3% BSA in TBST and detected using LI-COR IRdye labeled secondary antibodies (donkey anti-mouse 680/800, donkey anti-rabbit 680/800), diluted 1:10,000 in 3% BSA in TBST. Membranes were scanned on a LICOR Odyssey DLx scanner. Images were saved as .tif files and analyzed using the gel scanning plugin in FIJI (62).

### Immunohistochemical Staining.

Mouse brain striatum and olfactory epithelium were subjected to immunostaining following a previously established protocol (10.17504/protocols.io.bnwimfce). For the striatum, coronal sections spanning +1.2 to 0.0 mm anterior to posterior (AP) relative to bregma were used. Representative low-magnification images of the striatal sections are shown in *SI Appendix*, Fig. S1*A*. Frozen slides were thawed at room temperature for 15 min and then gently washed twice with PBS for 5 min each. Antigen retrieval was achieved by incubating the slides in 10 mM sodium citrate buffer pH 6.0, preheated to 95 °C, for 15 min.

### Expansion Microscopy of Olfactory Epithelium.

Expansion microscopy of the olfactory epithelium was performed as described (10.17504/protocols.io.6qpvry22ogmk/v1) to achieve ~4-5X isotropic expansion. Frozen olfactory epithelium sections were thawed at room temperature for 1 min, washed three times in PBS, and incubated for 2 h at RT in a monomer-fixative solution containing 0.7% PFA and 1% acrylamide in PBS to anchor proteins to the gel matrix. Sections were then embedded in a hydrogel composed of 19% sodium acrylate, 10% acrylamide, and 0.1% bis-acrylamide in PBS. Polymerization was initiated using ammonium persulfate and TEMED, first at 4 °C for 5 min and subsequently overnight at room temperature in a humidified chamber. Gels were denatured at 95 °C for 6 h in a buffer containing 50 mM Tris (pH 9.0), 200 mM NaCl, and 200 mM SDS. Gels were then fully expanded by repeated incubation in deionized water. Expanded gels were incubated overnight at 4 °C with goat anti-OMP (1:100) and mouse anti-acetylated tubulin (1:500), followed by overnight incubation at 4 °C with Alexa Fluor conjugated secondary antibodies (1:500) and DAPI. The gels were washed in PBS, fully expanded in deionized water and mounted on poly-D-lysine coated imaging dishes and imaged using the Zeiss LSM 900 confocal microscope to visualize olfactory sensory neuron cilia.

### Fluorescence In Situ Hybridization (FISH).

RNAscope fluorescence in situ hybridization was carried out as described [https://bio-protocol.org/exchange/preprintdetail?id=1423&type=3&searchid=EM1708992000021453&sort=5&pos=b; (11)]. The RNAscope Multiplex Fluorescent Detection Kit v2 (Advanced Cell Diagnostics) was utilized following the manufacturer’s instructions, employing RNAscope 3-plex Negative Control Probe (#320871) or Mm-Ptch1-C2 (#402811-C2), Mm-Gdnf (#421951), Mm-Nrtn-C2 (#441501-C2), Mm-Pvalb-C3 (#421931-C3), and Mm-Bdnf-C1 (#424821). The Mm-GDNF, Mm-Nrtn-C2, and Mm-Pvalb-C3 probes were diluted 1:30, 1:4, and 1:10, respectively, in dilution buffer consisting of 6× saline-sodium citrate buffer (SSC), 0.2% lithium dodecylsulfate, and 20% Calbiochem OmniPur Formamide.

### Microscope Image Acquisition.

All images were obtained using a Zeiss LSM 900 confocal microscope (Axio Observer Z1/7) coupled with an Axiocam 705 camera and immersion objective (Plan-Apochromat 63×/1.4 Oil DIC M27). The images for epithelial cells, astrocytes, and neurons were acquired at 0.4, 0.5, and 0.75 µm Z-sampling, respectively. The images were acquired using ZEN 3.4 (blue edition) software, and visualizations and analyses were performed using ImageJ Fiji (62), CellProfiler (63), and R (64).

### Quantification of Phospho-α-synuclein in Mouse Striatal Cholinergic Neurons and Analysis by Cilia Status.

Fluorescence microscopy z-stacks from wild-type and*Snca*^G51D/G51D^ mutant mouse brain tissues stained for pSNCA, AC3, ChAT, and DAPI were acquired and processed in FIJI/ImageJ using a custom FIJI macro (10.17504/protocols.io.5jyl8xjzdv2w/v1) to generate nonoverlapping 3-slice maximum-intensity projections across the full z-depth for each channel. All 3-slice projections derived from the same z-stack were treated as belonging to a single image dataset corresponding to one field of view. Projection images were imported into CellProfiler (63), where pSNCA-positive signal was segmented and restricted to only ChAT-positive cholinergic neurons. Integrated pSNCA intensity was measured for each ChAT-positive cholinergic neuron. A detailed protocol of the CellProfiler (63) pipeline is referenced here (10.17504/protocols.io.261ge13bwv47/v2).

For comparisons within the *Snca*^G51D/G51D^ mutant group, pSNCA measurements were grouped according to cilia status. Ciliary status (present or absent) was annotated (10.17504/protocols.io.bnwimfce) for each ChAT-positive neuron and linked to the corresponding pSNCA measurement from the same segmented cell. CellProfiler output tables were imported into R (64) for analysis. pSNCA distributions were visualized using histograms, plotted as the percentage of neurons per intensity bin.

### Quantification of Phospho-α-synuclein in Mouse Striatal Cholinergic and Medium Spiny Neurons.

Fluorescence microscopy z-stacks from *Snca*^G51D/G51D^ mutant mouse brain tissues stained for pSNCA, DARPP32, ChAT, and DAPI channels, were processed using the same custom FIJI/ImageJ macro described above (10.17504/protocols.io.5jyl8xjzdv2w/v1) to generate nonoverlapping 3-slice maximum-intensity projections across the z-axis. Projection images were imported into CellProfiler, where pSNCA-positive signal was segmented and cell-type masks were generated using DARPP32 and ChAT fluorescence to identify medium spiny neurons (MSNs) and ChAT-positive cholinergic neurons, respectively. Integrated pSNCA intensity was measured within each cell-type mask for every 3-slice projection. The same protocol described above (10.17504/protocols.io.261ge13bwv47/v2) was used for analysis, in which integrated pSNCA fluorescence intensities were summed per dataset separately for ChAT-positive and DARPP32-positive neurons and normalized to the corresponding total segmented cell mask area, yielding cell type–specific pSNCA intensity per unit area.

All scripts (macro and R) and the CellProfiler pipeline are available at 10.5061/dryad.4xgxd25r0.

### Statistical Analysis.

In addition to above-mentioned methods, all other statistical analysis was carried out using GraphPad Prism version 10.2.3 for Macintosh, GraphPad Software, Boston, MA, www.graphpad.com.

## Supplementary Material

Appendix 01 (PDF)

## Data Availability

All data have been deposited in DRYAD (https://doi.org/10.5061/dryad.4xgxd25r0) (65). All other data are included in the article and/or *SI Appendix*.
