# Supplementary material for: Selective loss of primary cilia and neurotrophic signaling in G51D α-synuclein mice highlights a common pathway to Parkinson’s disease
Source: Proc Natl Acad Sci U S A. 2026 Aug 10;123(33):e2619797123. doi: 10.1073/pnas.2619797123 (PMC13486547; doi:10.1073/pnas.2619797123)
Supplement: Supplementary file 1 [file pnas.2619797123.sapp.pdf]

PNAS

Supporting Information for

Selective loss of Primary Cilia and Neurotrophic Signaling in G51D α-Synuclein Mice Highlights a Common Pathway to Parkinson's Disease

Yu-En Lin, Ebsy Jaimon, YoungDoo Kim, Annabeth Loftman, Aaran Vijayakumaran, Benjamin D.W. Belfort, Claire Y. Chiang, Benjamin R. Arenkiel, Huda Y. Zoghbi, and Suzanne R. Pfeffer*

*Suzanne Pfeffer
Email: pfeffer@stanford.edu

This PDF file includes:

Figures S1 to S4
Key Resources Table

Figures

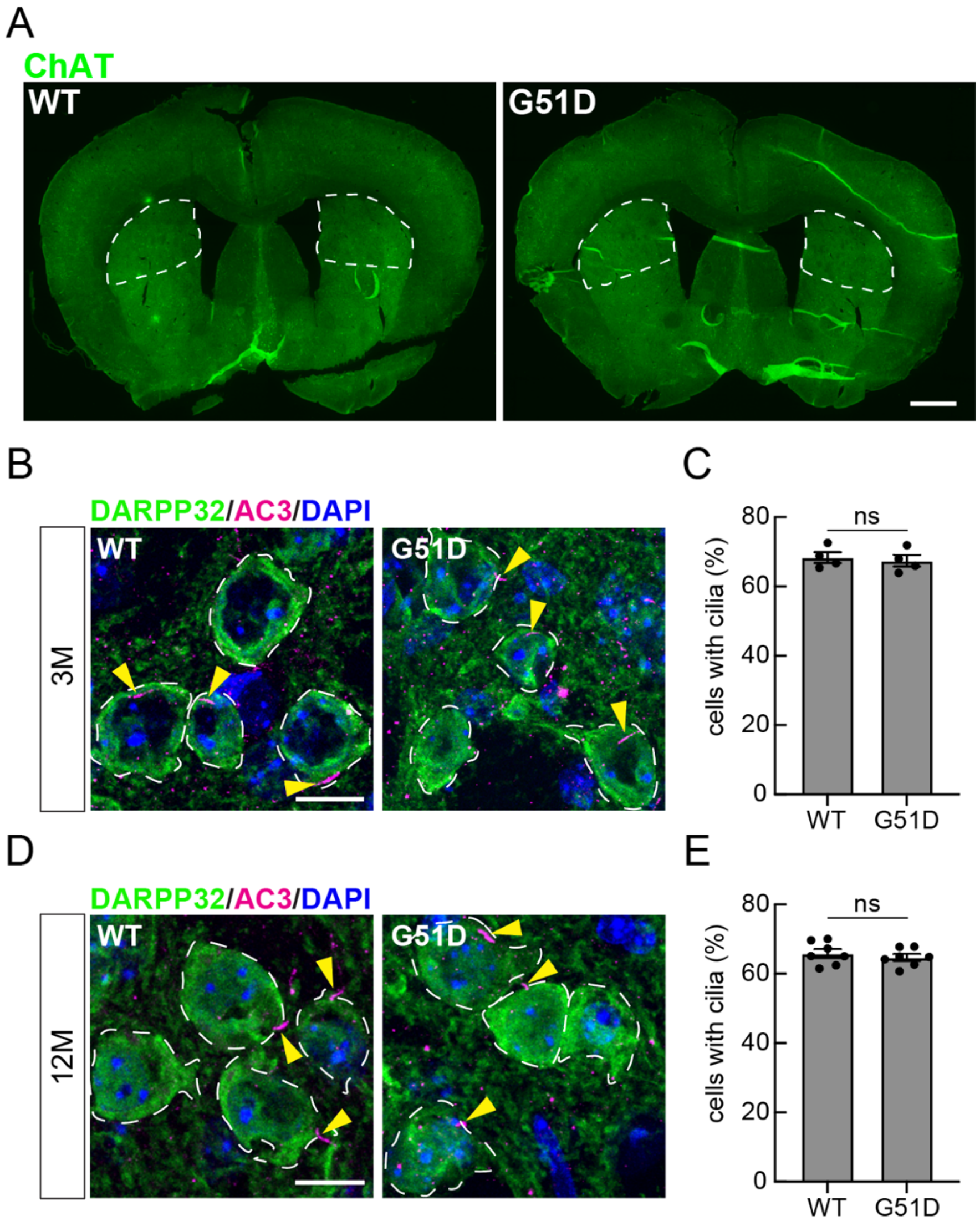

Fig. S1. The *Snca*$^{G51D/G51D}$ mutation does not induce cilia defects in predominant spiny projection neurons of the mouse dorsal striatum.
(A) Representative low-magnification images of coronal striatal sections from 12-month-old wild-type and *Snca*$^{G51D/G51D}$ mice immunostained for choline acetyltransferase (ChAT, green). The dashed lines outline the dorsal striatum, which is the region of interest analyzed in this study. Scale bar, 100 µm. **(B and D)** Representative images of dorsal striatal sections from 3- and 12-month-old wild-type and *Snca*$^{G51D/G51D}$ mice. Spiny projection neurons (SPNs) labeled with anti-DARPP32 antibody (green); primary cilia labeled with anti-AC3 antibody (magenta; yellow arrowheads). Nuclei were labeled with DAPI (blue). Scale bars, 10 µm. **(C and E)** Quantitation of DARPP32^{+} neurons containing a cilium in 3- and 12-month-old wild-type and *Snca*$^{G51D/G51D}$ mice, respectively. Values represent mean ± SEM from four (3-month-old) or seven (12-month-old) individual mice per group, with two to three sections analyzed per mouse. More than 306 and 300 DARPP32^{+} SPNs were scored per mouse in the 3- and 12-month group (mean ± SD of 329 ± 17 and 335 ± 25, respectively). Statistical significance was determined using an unpaired Student's *t* test. Differences were not significant (ns; $p = 0.7093$ at 3 months and $p = 0.4837$ at 12 months).

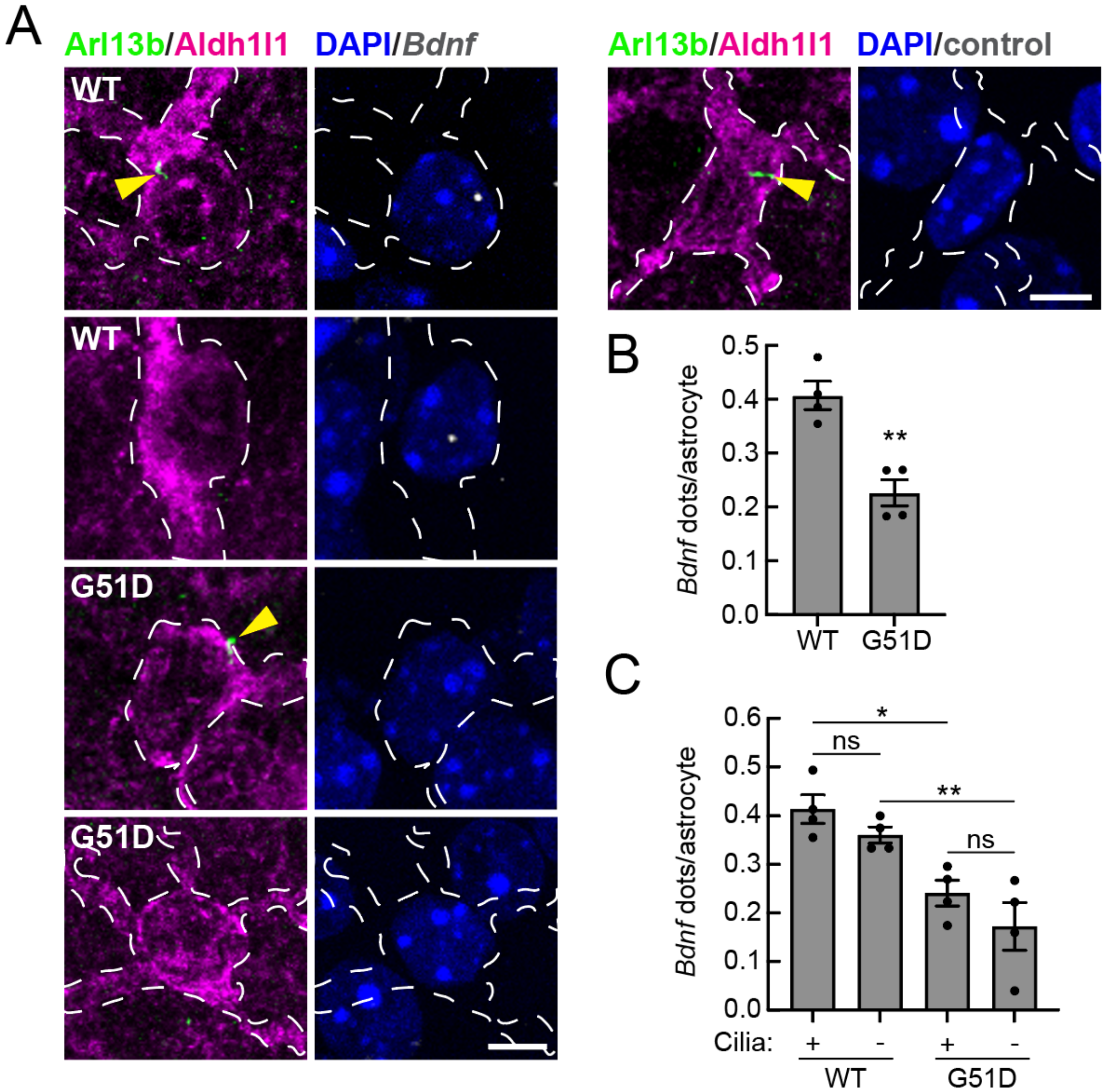

Fig. S2. Reduced BDNF expression in striatal astrocytes of *Snca*$^{G51D/G51D}$ mice.
(A) Representative confocal immunofluorescence images of astrocytes from 12-month-old wild-type and *Snca*$^{G51D/G51D}$ mice. Astrocytes were labeled with anti-Aldh1l1 antibody (magenta), and astrocytic primary cilia were labeled with anti-Arl13B antibody (green; yellow arrowheads), and nuclei were labeled with DAPI (blue). Sections were subjected to RNAscope in situ hybridization using a *Bdnf* RNA probe (white dots). Images of a control astrocyte processed without the RNAscope probe are shown above panel (B), at the top right. Scale bar, 5 µm. **(B)** Quantitation of the average number of *Bdnf* dots per Aldh1l1^{+} astrocyte. **(C)** Quantitation of the average number of *Bdnf* dots segregated by ciliation status. Values represent mean ± SEM from four mice per group, with two to three sections analyzed per mouse. More than 79 Aldh1l1^{+} astrocytes were scored per mouse (mean ± SD = 101 ± 14). Statistical significance was determined using an unpaired Student's *t* test or one-way ANOVA with Tukey's post hoc test. **p = 0.0024 (wild-type versus G51D); *p = 0.0128 (ciliated wild-type versus ciliated G51D); **p = 0.0072 (unciliated wild-type versus unciliated G51D). Differences were not significant (ns; p = 0.6649 for ciliated wild-type versus unciliated wild-type; p = 0.4751 for ciliated G51D versus unciliated G51D).

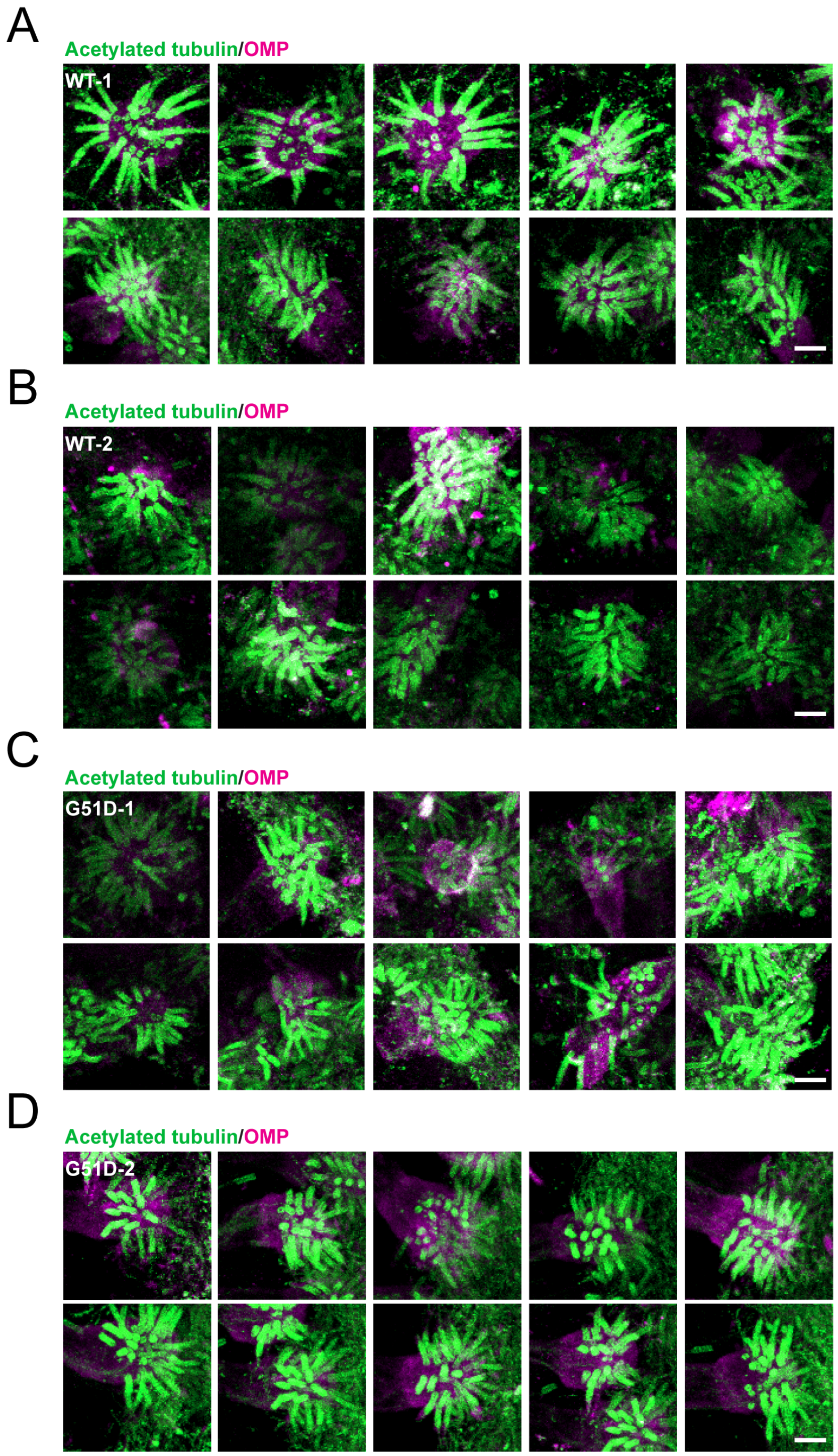

Fig. S3. Representative confocal images of the OSN cilia of 12 month-old wild-type and *Snca*$^{G51D/G51D}$ mice visualized using expansion microscopy.
OSNs were labeled with anti-OMP antibody (magenta), and cilia with anti-Acetylated alpha-tubulin antibody (green). All images are from 12-month-old animals. **(A)** Wild-type animal 1(WT-1), **(B)** Wild-type animal 2 (WT-2), **(C)** *Snca*$^{G51D/G51D}$ animal 1 (G51D-1). **(D)** *Snca*$^{G51D/G51D}$ animal 2 (G51D-2). Scale bar, 1 μm.

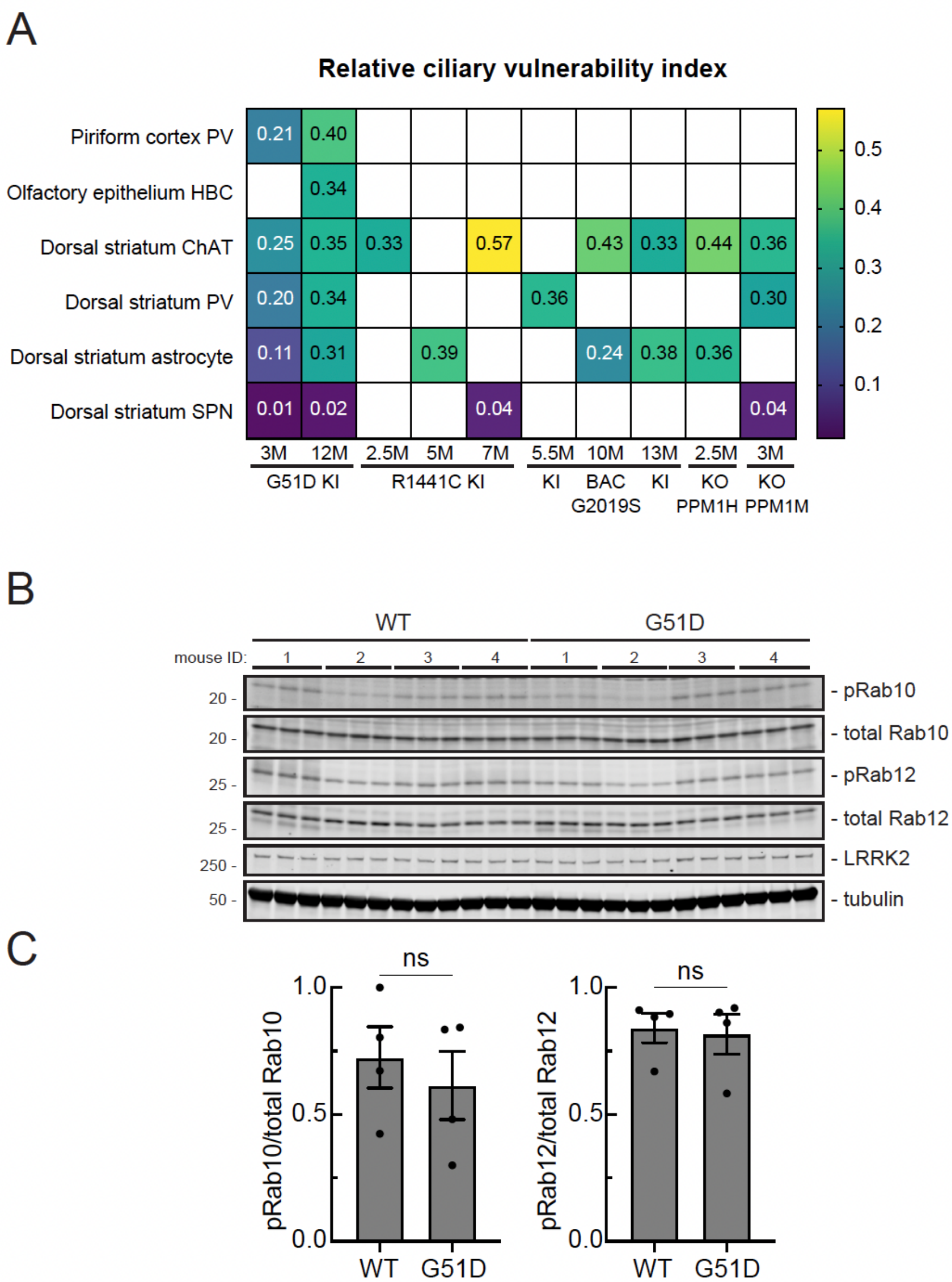

Fig. S4. Cell type–specific ciliary vulnerability occurs without detectable changes in Rab10/12 phosphorylation in whole-brain tissue of *Snca*$^{G51D/G51D}$ mice.
(A) Relative ciliary vulnerability index for PV interneurons in the piriform cortex, horizontal basal cells (HBCs) in the olfactory epithelium, and cholinergic interneurons (ChAT^{+}), PV interneurons, spiny projection neurons (SPN), and astrocytes in the dorsal striatum, measured at the indicated ages and compiled across *Snca*$^{G51D/G51D}$ knock-in and LRRK2 pathway mutant mouse lines, highlighting cell type–, region–, and age–specific susceptibility of primary cilia. The index was calculated as 1 − (ciliation rate of a given cell type in the mutant / ciliation rate of the corresponding cell type in the control), such that higher values and brighter colors indicate greater ciliary vulnerability. **(B)** Immunoblot analysis of 9-month-old wild-type (WT) and *Snca*$^{G51D/G51D}$ mouse whole brain lysate. Four mice were analyzed per genotype (mouse ID 1-4). Mass is shown on the left in kDa; antigens are indicated at the right. **(C)** Quantitation of phosphoRab10 (pRab10)/total Rab10 and phosphoRab12 (pRab12)/total Rab12 levels from immunoblots in (B). Values were normalized to 1.0 for the highest WT level, and each dot represents the average of two independent replicates from one mouse.

Table S1. Key Resource Table

RESOURCE TYPE	RESOURCE NAME	SOURCE	IDENTIFIER	NEW/ REUSE	ADDITIONAL INFORMATION
Genetic reagent (Mus musculus)	B6.$Snca^{G51D/G51D}$	Generated by and collaborated with Dr. Huda Zoghbi	Kim et al., Proc. Natl. Acad. Sci. U.S.A. 121 (39) e2406479121,https://doi.org/10.1073/pnas.2406479121 (2024).	REUSE	C57BL/6J; $Snca^{G51D/G51D}$ MGI:8319110
Antibody	anti-alpha tubulin (mouse monoclonal)	Santa Cruz Biotechnology	sc-32293 (RRID: AB_628412)	REUSE	(1:4000)
Antibody	anti-LRRK2 (mouse monoclonal)	Antibodies Incorporated/NeuroMab	N241A/34 (RRID: AB_10675136)	REUSE	(1:1000)
Antibody	anti-Rab10 pThr73 (rabbit monoclonal)	Abcam Inc.	ab230261 (RRID: AB_2811274)	REUSE	(1:1000)
Antibody	anti-Rab10 (mouse monoclonal)	Abcam Inc.	ab104859 (RRID: AB_10711207)	REUSE	(1:1000)
Antibody	anti-Rab12 pS106 (rabbit monoclonal)	Abcam Inc.	ab256487 (RRID: AB_2884880)	REUSE	(1:1000)
Antibody	anti-Rab12 (mouse monoclonal)	Santa Cruz Biotechnology	sc-515613 (RRID: AB_3101762)	REUSE	(1:500)
Antibody	IRDye 800CW Donkey anti-Rabbit IgG	LI-COR	926-32213 (RRID: AB_621848)	REUSE	(1:10,000)
Antibody	IRDye 680RD Donkey anti-Rabbit IgG	LI-COR	926-68073 (RRID: AB_10954442)	REUSE	(1:10,000)
Antibody	IRDye 800CW Donkey anti-Mouse IgG	LI-COR	926-32212 (RRID: AB_621847)	REUSE	(1:10,000)
Antibody	IRDye 680RD Donkey anti-Mouse IgG	LI-COR	926-68072 (RRID: AB_10953628)	REUSE	(1:10,000)
Antibody	anti-Choline Acetyltransferase (goat polyclonal)	Millipore	AB144P-1ML (RRID:AB_2079751)	REUSE	(1:200)
Antibody	anti-Adenylate cyclase III (rabbit polyclonal)	EnCOR	RPCA-ACIII (RRID:AB_2572219)	REUSE	(1:10,000)
Antibody	anti-Parvalbumin (guinea pig polyclonal)	Synaptic systems	#195004 (RRID:AB_2156476)	REUSE	(1:1000)
Antibody	anti-Arl13b (mouse monoclonal)	Neuromab	N295B/66 RRID:AB_2877361	REUSE	(1:1000)
Antibody	anti-ALDH1L1 (rabbit polyclonal)	Proteintech	17390-1-AP RRID:AB_2878401	REUSE	(1:250)
Antibody	anti-Adenylate cyclase III (chicken polyclonal)	EnCOR	CPCA-ACIII (RRID:AB_2744500)	REUSE	(1:5000)

Antibody	anti-DARPP32 (rabbit monoclonal)	Cell Signaling Technology	#2306S (RRID:AB_823479)	REUSE	(1:1000)
Antibody	anti-DARPP32 (guinea pig polyclonal)	Synaptic Systems	#328005 (RRID:AB_2721086)	REUSE	(1:1000)
Antibody	anti-phosphopho-α-synuclein (Ser129) (clone: D1R1R) (rabbit monoclonal)	Cell Signaling Technology	#87281 (RRID:AB_279886)	REUSE	(1:1000)
Antibody	Anti-p63 (rabbit monoclonal)	Abcam Inc.	Ab124762 (RRID: AB_10971840)	REUSE	(1:6000)
Antibody	Anti-OMP (goat polyclonal)	FUJIFILM Wako Pure Chemical Corporation	#54-10001-WAKO (RRID:AB_664696)	REUSE	(1:200 for IHC and 1:100 for expansion microscopy)
Antibody	Anti-Acetylated tubulin (mouse monoclonal)	Millipore	#T6793 (RRID:AB_477585)	REUSE	(1:1000 for IHC and 1:500 for expansion microscopy)
Antibody	H+L Donkey anti-Goat AF 488	Life Technologies	A11055 (RRID:AB_2534102)	REUSE	(1:2000)
Antibody	H+L Donkey anti-Rabbit AF 568	Life Technologies	A10042 (RRID:AB_2534017)	REUSE	(1:2000)
Antibody	H+L Donkey anti-guinea pig Alexa 647	Jackson ImmunoResearch	#706-605-148 (RRID:AB_2340476)	REUSE	(1:2000)
Antibody	H+L Donkey anti-mouse AF 647	Life Technologies	A31571 (RRID:AB_162542)	REUSE	(1:2000)
Antibody	H+L Donkey anti-mouse AF 488	Life Technologies	A32766 (RRID:AB_2762823)	REUSE	(1:2000)
Antibody	H+L Donkey anti-chicken Alexa 488	Jackson ImmunoResearch	#703-545-155 (RRID:AB_2340375)	REUSE	(1:2000)
Antibody	H+L Donkey anti-rabbit AF 647	Life Technologies	A31573 (RRID:AB_2536183)	REUSE	(1:2000)
Antibody	H+L Donkey anti-Goat AF 568	Life Technologies	A11057 (RRID:AB_2534104)	REUSE	(1:2000)
Antibody	H+L Donkey anti-guinea pig CF™ 488	Sigma-Aldrich	SAB4600033 (RRID:AB_2890881)	REUSE	(1:2000)

Chemical, peptide, or recombinant protein	Bio-Rad Protein Assay Dye Reagent Concentrate	Bio-Rad	5000006	REUSE	
Chemical, peptide, or recombinant protein	cOmplete EDTA-free protease inhibitor cocktail	Roche	11873580001	REUSE	
Chemical, peptide, or recombinant protein	PhosSTOP phosphatase inhibitor cocktail	Roche	4906837001	REUSE	
Chemical, peptide, or recombinant protein	Microcystin-LR	Sigma	475815-M	REUSE	
Chemical, peptide, or recombinant protein	DAPI	Sigma	D9542	REUSE	
Critical commercial assay	Trans-Blot Turbo RTA Midi 0.2 μm Nitrocellulose Transfer Kit	Bio-Rad	1704271	REUSE	
Critical commercial assay	RNAscope Multiplex Fluorescent Reagent Kit v2	Advanced Cell Diagnostics	#323100	REUSE	
Critical commercial assay	RNAscope Multiplex Fluorescent Reagent Kit v2	Advanced Cell Diagnostics	#323100	REUSE	
Critical commercial assay	RNAscope Probe-Mm-Ptch1-C2	Advanced Cell Diagnostics	#402811-C2	REUSE	1:5
Critical commercial assay	RNAscope Probe-Mm-Gdnf	Advanced Cell Diagnostics	#421951	REUSE	1:30
Critical commercial assay	RNAscope Probe-Mm-Nrtn-C2	Advanced Cell Diagnostics	#441501-C2	REUSE	1:4
Critical commercial assay	RNAscope Probe-Mm-Pvalb-C3	Advanced Cell Diagnostics	#421931-C3	REUSE	1:10
Critical commercial assay	RNAscope Probe-Mm-Bdnf	Advanced Cell Diagnostics	#424821	REUSE	

Critical commercial assay	OPAL 690 REAGENT PACK	Akoya Biosciences	FP1497001KT	REUSE	
Critical commercial assay	OPAL 570 REAGENT PACK	Akoya Biosciences	FP1488001KT	REUSE	
Dataset	Selective loss of Primary Cilia and Neurotrophic Signaling in G51D α-Synuclein Mice Highlights a Common Pathway in Parkinson's Disease	Dryad	https://doi.org/10.5061/dryad.4xgxd25r0	NEW	The dataset includes raw microscopy images (.czi), immunoblot images (.tif), quantitative analyses in GraphPad Prism format (.pzf), tabulated data files (.csv), and analysis scripts, including FIJI/ImageJ macros, R scripts, and CellProfiler pipelines.
Protocol	Protein extraction and western blot (mouse tissues)	protocols.io	dx.doi.org/10.17504/protocols.io.sqgedtw	REUSE	
Protocol	Analysis of Primary Cilia in Rodent Brain By Immunofluorescence Microscopy	protocols.io	dx.doi.org/10.17504/protocols.io.bnwimfce	REUSE	
Protocol	A 4-5X expansion microscopy protocol for high-resolution imaging of olfactory sensory neuron cilia in mouse olfactory epithelium	protocols.io	dx.doi.org/10.17504/protocols.io.6qpvry22ogmk/v1	New	
Protocol	Fluorescence in situ hybridization (FISH)	Bioprotocol	https://bio-protocol.org/exchange/preprintdetail?id=1423&type=3&searchid=EM1708992000021453&sort=5&pos=b	REUSE	
Protocol	An ImageJ/FIJI Preprocessing Workflow for Multi-Series Confocal Microscopy Datasets Prior To CellProfiler Analysis	protocols.io	dx.doi.org/10.17504/protocols.io.5jyl8xjzdv2w/v1	NEW	
Protocol	A CellProfiler Pipeline for Quantification of p-SNCA in Mouse Striatal	protocols.io	dx.doi.org/10.17504/protocols.io.261ge13bwv47/v2	NEW	

	Cholinergic and Medium Spiny Neurons V.2				
Software/code	FIJI	PMID:29187165	RRID:SCR_002285	REUSE	
Software/code	Graphpad Prism	Prism 10 version 10.2.3	RRID: SCR_002798	REUSE	
Software/code	ZEN	Zeiss ZEN Microscopy Software	RRID:SCR_013672	REUSE	
Software/code	R	http://www.r-project.org/	RRID:SCR_001905	REUSE	
Software/code	CellProfiler version 4.2.6	PMID: 34507520	RRID:SCR_007358	REUSE	
